# First-in-human anti-Trop2 CAR-T cell therapy in refractory INI-1/BRM-deficient sinonasal carcinoma: A case report

**DOI:** 10.1016/j.omton.2026.201366

**Published:** 2026-10-14

**Authors:** Guangfei Li, Yuting Lai, Liang Wang, Hongjia Zhu, Min Chen, Jifang Wang, Taomin Huang, Liqing Kang, Nan Xu, Minghao Li, Xiaoyan Lou, Zhou Yu, Wei Wang, Minghao Fan, Jiali Wu, Haitao Wu, Lei Yu, Peijie He, Hongmeng Yu, Jian Chen

**Affiliations:** 1 ENT Institute and Department of Otorhinolaryngology, Eye & ENT Hospital, Fudan University, Shanghai 200031, China; 2 School of Pharmacy, East China Normal University, Shanghai 200062, China; 3 Shanghai Unicar-Therapy Bio-medicine Technology Co., Ltd, Shanghai 200000, China; 4 Department of Nursing, Eye & ENT Hospital, Fudan University, Shanghai 200031, China; 5 Department of Pharmacy, Eye & ENT Hospital, Fudan University, Shanghai 200031, China; 6 Shanghai Key Laboratory of Gene Editing and Cell Therapy for Rare Diseases, Fudan University, Shanghai 200031, China

**Keywords:** chimeric antigen receptor, sinonasal carcinoma, INI-1 deficient, cytokine release syndrome, hepatotoxicity

## Abstract

INI-1-deficient sinonasal carcinoma is an aggressive malignancy with a dismal prognosis and no established salvage therapies. While trophoblast cell-surface antigen 2 (Trop2) is a promising immunotherapeutic target, its clinical utility in sinonasal cancer remains unexplored. We report the first-in-human application of anti-Trop2 CAR-T cell therapy in a 61-year-old male with refractory INI-1/BRM-deficient sinonasal carcinoma. Following lymphodepletion, the CAR-T cells demonstrated robust *in vivo* expansion, peaking within 14 days and coinciding with transient cytokine elevation. Toxicities were manageable but included grade 2 cytokine release syndrome and grade 3 hepatotoxicity that required multiple sessions of plasma exchange. Notably, MRI at day 78 post-infusion revealed dramatic tumor regression with extensive necrosis and liquefaction, which correlated with the complete resolution of intractable orbital pain. This single-case observation provides preliminary proof-of-concept for the safety and anti-tumor activity of Trop2-targeted CAR-T cells in refractory sinonasal malignancies, though further studies are needed to confirm durability and generalizability.

## Graphical abstract

**Figure:**
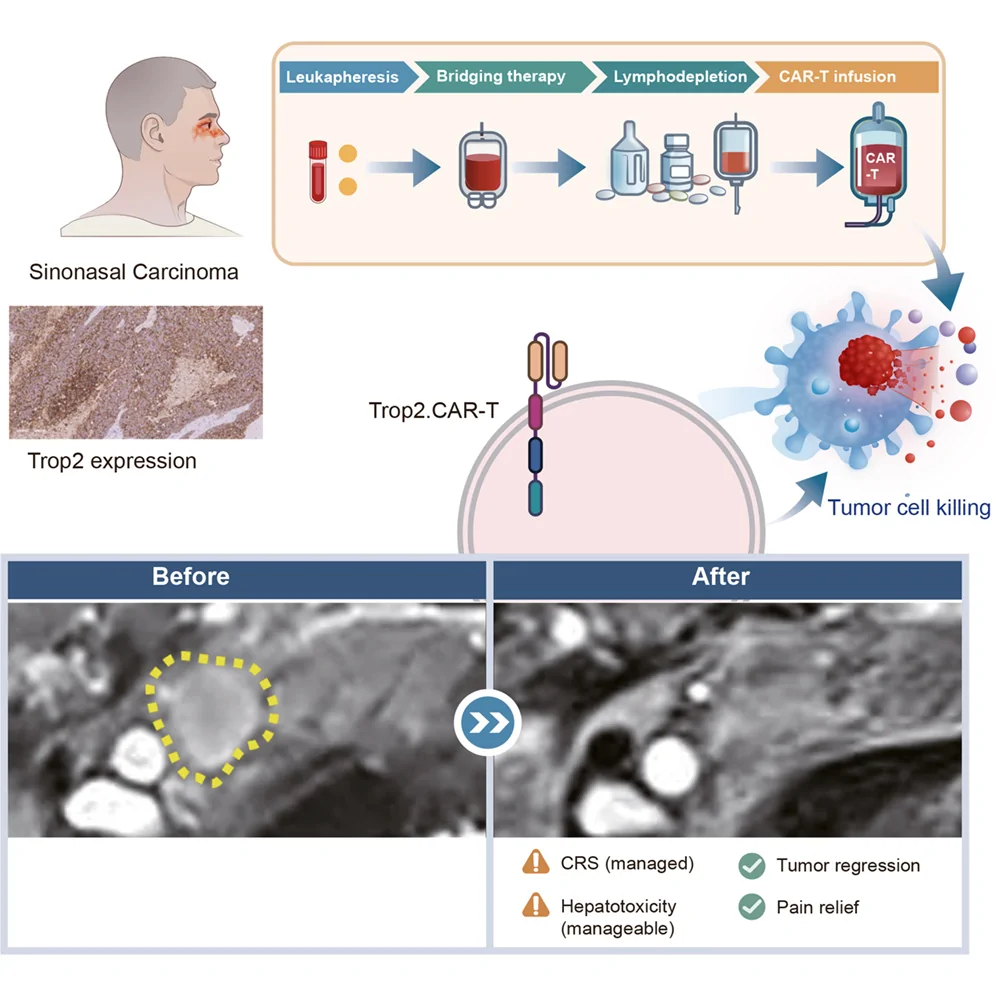

## Teaser

This first-in-human case report describes anti-Trop2 CAR T cell therapy in refractory INI-1/BRM-deficient sinonasal carcinoma, a disease with poor prognosis. Treatment produced transient Trop2 CAR-T cell expansion and tumor regression, accompanied by cytokine release syndrome and hepatotoxicity. These findings support further clinical evaluation of Trop2-targeted CAR T cells.

## Introduction

Sinonasal malignancies, though accounting for only 3%–5% of all head and neck tumors, represent a formidable clinical challenge characterized by aggressive local invasion and profound resistance to conventional multimodal therapies.^1^^,^^2^ Within this heterogeneous spectrum, carcinomas driven by the deficiency of the switch/sucrose non-fermentable (SWI/SNF) chromatin, most frequently through the biallelic loss of INI-1 (SMARCB1) and/or BRM (SMARCA2), exhibit an undifferentiated histology and an exceptional lydismalprognosis.^3^^,^^4^ For patients with recurrent or metastatic disease, no standardized targeted or immunotherapeutic regimens have been established, and the 3-year overall survival rate remains a modest 51.8%.^5^

Chimeric antigen receptor (CAR)-T cell therapy offers a potential paradigm shift for such intractable solid tumors.^6^ A pivotal requirement for this cellular approach is the identification of tumor-associated antigens that are stably and abundantly expressed on malignant cells while maintaining an acceptable safety profile.^7^ Trophoblast cell surface antigen 2 (Trop2), a transmembrane glycoprotein overexpressed across a wide range of epithelial cancers, has emerged as a clinically validated target that drives tumor proliferation and therapeutic resistance.^8^^,^^9^ While Trop2-directed antibody-drug conjugates have entered clinical practice, anti-Trop2 CAR-T cells are only recently being explored, with early-phase trials suggesting promising activity in head and neck squamous cell carcinoma.^10^^,^^11^ However, the clinical utility of Trop2-targeted cellular therapy in sinonasal malignancies remains unexplored.

Here, we report the first clinical application of autologous anti-Trop2 CAR-T cell therapy in a patient with treatment-refractory sinonasal carcinoma harboring concomitant INI-1/BRM deficiency and high Trop2 expression. We detail the therapeutic feasibility, robust *in vivo* expansion, and remarkable tumor regression achieved in this ultra-rare and aggressive disease entity, providing preliminary proof of concept for Trop2 as a viable therapeutic vulnerability in sinonasal malignancies.

### Patient details and clinical findings

In March 2024, a 61-year-old male presented with right nasal obstruction and blood-tinged discharge and underwent endoscopic sinonasal tumor resection. Postoperative pathology confirmed “INI-1-deficient carcinoma of the right nasal cavity.” He subsequently received two cycles of chemotherapy in April 2024, followed by a second radical resection of the right sinonasal tumor in June 2024. In July 2024, he underwent radiotherapy (50 fractions) with concurrent chemotherapy. Despite this aggressive multimodal approach, MRI on March 21, 2025, revealed a new soft tissue mass in the right surgical cavity and ethmoid sinus region, indicating local recurrence. On March 31, 2025, the patient underwent a comprehensive salvage surgery at our center, including endoscopic sinonasal tumor resection, endoscopic skull base lesion resection, complete sinusotomy, endoscopic dacryocystorhinostomy, and right orbital decompression. Postoperatively, he received two additional cycles of chemotherapy from April to June 2025. However, the disease continued to progress, and the patient was clinically diagnosed with advanced refractory relapsed INI-1-deficient sinonasal carcinoma. A repeat biopsy during this period confirmed SWI/SNF complex deficiency with concomitant loss of INI-1 and BRM, along with extensive bone invasion. Immunohistochemistry showed strong and diffuse Trop2 expression (Figure 1A), providing the rationale for Trop2-targeted therapy. Additional methodological details are provided in the supplemental information.

**Figure 1:**
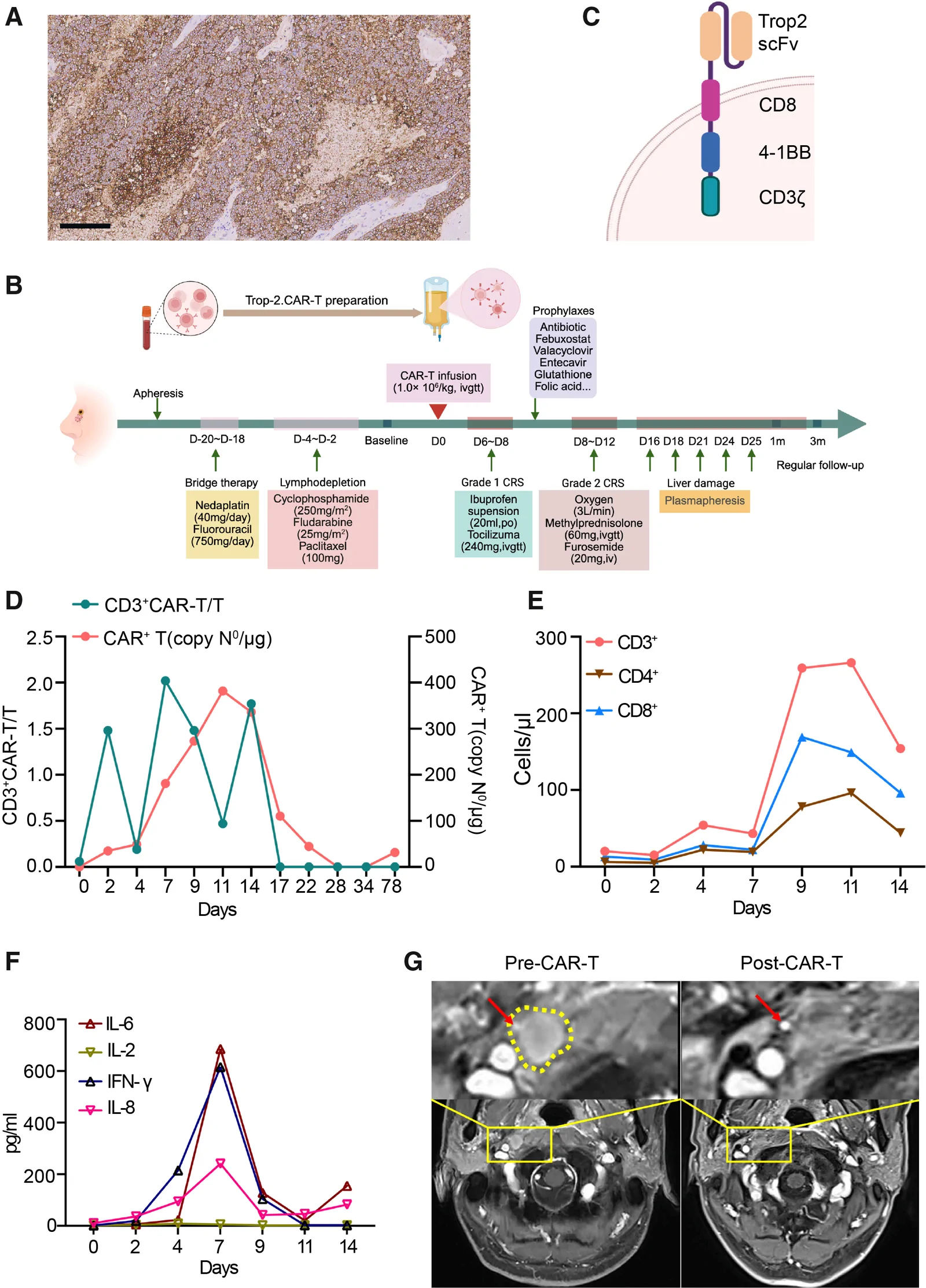
Treatment schema, CAR-T expansion, and anti-tumor response following Trop-2 CAR-T therapy (A) Tumor immunohistochemistry confirming high Trop-2 expression. (B) Schematic overview of the treatment procedures. (C) CAR construct comprising Trop-2 scFv, CD8 hinge/transmembrane domain, 4-1BB costimulatory domain, and CD3ζ signaling domain. (D) Peripheral expansion of CAR-T cells, shown as CD3^+^CAR-T frequency and transgene copy number, peaking within the first 2 weeks post-infusion. (E) Dynamics of circulating T cell subsets (CD3^+^, CD4^+^, and CD8^+^), demonstrating transient expansion consistent with CAR-T proliferation. (F) Serum cytokine kinetics (IL-6, IL-2, IFN-γ, and IL-8), with a sharp peak around day 7 corresponding to CRS onset. (G) MRI evaluation showing marked tumor regression at day 78 compared with baseline. (Scale bars, 50 μm).

The patient was enrolled in the first cohort of an investigator-initiated phase 1 clinical trial (NCT06614686) employing a standard 3 + 3 dose-escalation design. As the first-treated patient, he received the lowest planned dose level (1.0 × 10^6^ cells/kg), and thus this report reflects the initial clinical experience with this anti-Trop2 CAR-T product. The trial was approved by the institutional ethics committee.

After obtaining informed consent, the patient underwent leukapheresis on July 10, 2025 (Figure 1B). During CAR-T manufacturing (July 10–August 4), the patient received bridging chemotherapy with nedaplatin plus fluorouracil from July 15 to July 17, 2025, to stabilize tumor burden (nedaplatin 40 mg/day and fluorouracil 0.75 g/day, both administered intravenously once daily for 3 consecutive days). A single intravenous dose of 1.0 × 10^6^ anti-Trop2 CAR-T cells/kg was infused on August 4, 2025 (Figure 1C). The infusion was followed by a transient *in vivo* expansion of the CAR-T cells (Figure 1D). Notably, both CD4^+^ and CD8^+^ T cell subsets expanded, with a predominant rise in the CD8^+^ cytotoxic compartment (Figure 1E). The cellular expansion coincided with a transient elevation of pro-inflammatory cytokines, including IL-6, IFN-γ, and IL-8, which peaked around day 7 (Figure 1F).

Clinically, notwithstanding the limited longitudinal persistence of CAR-T cells in the peripheral circulation, the patient achieved a rapid and profound therapeutic response. Within only one week of infusion, there was a significant and rapid relief of the previously intractable orbital pain, which completely resolved by the second week. This early symptomatic improvement was further substantiated by definitive radiological evidence at day 78 post-treatment. MRI evaluation revealed a remarkable response characterized by substantial tumor regression, extensive central necrosis, and liquefaction. Notably, the relief of the tumor’s mass effect led to the clear demarcation of previously obscured vascular structures, providing robust evidence of a potent anti-tumor effect that persisted well beyond the peak of systemic CAR-T expansion (Figure 1G). No additional anti-cancer therapies (chemotherapy, radiotherapy, or other immunotherapies) were administered after CAR-T cell infusion. The patient received only supportive care and management of adverse events as described in the following text. Therefore, the observed tumor response is primarily attributed to CAR-T cell therapy.

The treatment was associated with manageable toxicities. On day 7, coinciding with peak cytokine levels and CAR-T expansion, the patient developed a high fever and subsequent hypoxia with pulmonary edema. These symptoms, consistent with grade 2 cytokine release syndrome (CRS), were promptly reversed following treatment with tocilizumab, corticosteroids, and supportive diuretics. A self-limiting diffuse erythematous rash appeared on day 2 and resolved by day 14 (Figure 2A). An oral fungal infection observed on day 12 was successfully managed with antifungal therapy (Figure 2B). Hepatotoxicity was the most significant adverse event. Liver enzymes and bilirubin rose from day 8, reaching grade 3 by day 13 (Figures 2C and 2D). Despite hepatoprotective agents, the patient required multiple sessions of plasma exchange (five sessions over two weeks) to manage progressive hyperbilirubinemia and elevated transaminases. Liver function eventually stabilized without progressing to liver failure, but recovery was gradual. Hematologic recovery followed the expected nadir after lymphodepletion (Figure 2E). Phenotypic analyses during *ex vivo* expansion showed tonic signaling (Figure 2F) and increased exhaustion markers (PD-1, TIM-3, and LAG-3) by day 14 (Figure 2G), suggesting antigen-independent tonic signaling that may have limited *in vivo* persistence.

**Figure 2:**
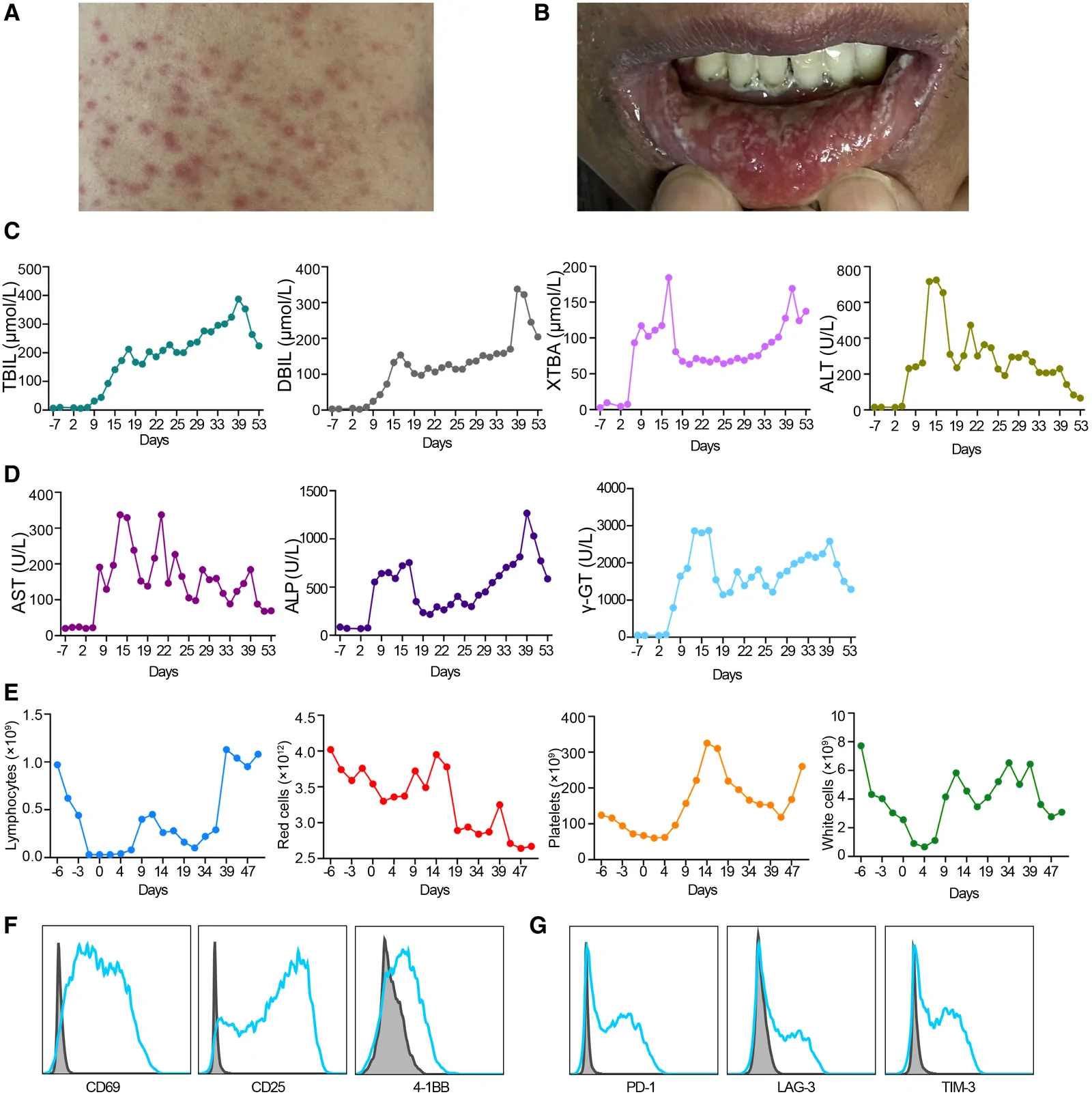
Manageable toxicities and dynamic immune-related adverse events following CAR-T therapy (A) Diffuse cutaneous rash observed post-infusion. (B) Oral mucosal ulceration as a manifestation of treatment-related toxicity. (C) Liver function parameters (TBIL, DBIL, TBA, and ALT) demonstrating progressive hepatotoxicity after CAR-T infusion. (D) Additional hepatic markers (AST, ALP, and γ-GT) confirming mixed hepatocellular-cholestatic injury. (E) Longitudinal blood counts showing post-lymphodepletion cytopenias followed by gradual hematologic recovery. (F) Flow cytometric analysis showing the tonic signaling of anti-Trop2 CAR-T cells during *ex vivo* expansion in the absence of antigen stimulation on day 14. (G) Increased expression of exhaustion markers (PD-1, TIM-3, and LAG-3) in anti-Trop2 CAR-T cells during *ex vivo* expansion on day 14.

## Discussion

This single-case report provides initial clinical evidence that anti-Trop2 CAR-T therapy can induce rapid tumor regression in refractory INI-1/BRM-deficient sinonasal carcinoma. The response is notable given the aggressive nature of this malignancy and the failure of multiple prior modalities. Trop2 is validated as a target by antibody-drug conjugates,^12^^,^^13^ and our observation extends this to cellular immunotherapy. However, as a single case with short follow-up, no definitive conclusions on efficacy can be drawn.

Safety remains a key concern. Grade 2 CRS and grade 3 hepatotoxicity occurred, the latter required multiple plasma exchange sessions, highlighting the need for close monitoring. Liver injury may be multifactorial, including cytokine-mediated inflammation or on-target/off-tumor effects from low-level Trop2 expression in biliary or hepatic tissues.^14^^,^^15^ These events were reversible with standard management, suggesting acceptable tolerability at this dose.

A notable finding is the dissociation between robust early tumor necrosis and limited CAR-T persistence. The patient achieved significant tumor reduction by day 78, yet CAR-T cells were barely detectable after day 17. This limited persistence may be due to tonic signaling-induced exhaustion, as our *ex vivo* data show spontaneous upregulation of activation and exhaustion markers without antigen stimulation (Figures 2F and 2G). Previous work has linked CAR tonic signaling to electrostatic interactions of the single-chain variable fragment (scFv), quantified by the positively charged patches (PCP) score.^16^^,^^17^ Although this CAR has a PCP score of 33 (below the optimal ∼40), it exhibited high tonic signaling, suggesting non-electrostatic mechanisms such as scFv stability or framework integrity.^18^^,^^19^^,^^20^^,^^21^ Engineering improvements are needed to reduce tonic signaling and enhance persistence.

In conclusion, this first report of anti-Trop2 CAR-T therapy in INI-1/BRM-deficient sinonasal carcinoma demonstrates feasibility and a promising initial response but also highlights the challenge of limited CAR-T persistence. Prospective trials with larger cohorts and modified CAR designs are required to assess long-term efficacy and safety.

## Data and code availability

Detailed preclinical validation of this CAR construct is available in a companion manuscript currently under review. These data can be made available to the editor and reviewers confidentially if required.

## Acknowledgments

We thank all the participants for their generous support for the current study. This study was supported by the Shanghai Hospital Development Center Foundation (SHDC12025122), the 10.13039/501100001809National Natural Science Foundation of China (82471150 to J.C.), the 10.13039/501100013105Shanghai Rising-Star Program (23QA1400900 to J.C.), China University Industry-Academia-Research Innovation Fund (2024DR033 to P.H.), as well as the Shanghai Clinical Research Center for Cell Therapy (23J41900100). This study was approved by the Institutional Review Board/Ethics Committee of Eye & ENT Hospital, 10.13039/501100003347Fudan University (approval no. 2024145). The trial is registered at ClinicalTrials.gov (NCT06614686). Written informed consent was obtained from the patient for participation in the study. A copy of the written informed consent is available for review by the editorial office upon request.

## Author contributions

The study was designed by P.H., H.Y., and J.C.; clinical data and patient care were provided by J. Wang., T.H., P.H., H.Y., J.C., and L.Y.; formal analyses were performed by G.L., Y.L., L.W., H.Z., and M.C.; figures were created by G.L., Y.L., and H.Z.; data were interpreted by G.L., Y.L., L.W., H.Z., M.C., J. Wang, T.H., L.K., N.X., M.L., X.L., Z.Y., W.W., M.F., J. Wu, L.Y., P.H., H.Y., and J.C.; resources were provided by J. Wang, T.H., L.K., N.X., M.L., X.L., Z.Y., W.W., M.F., J. Wu, L.Y., P.H., H.Y., and J.C.; original draft was written by G.L., Y.L., L.W., H.Z., M.C., P.H., H.Y., and J.C.; all authors critically reviewed and edited the manuscript; the study was supervised by P.H., H.Y., L.Y., and J.C.; the guarantors of the study are P.H., H.Y., and J.C.

## Declaration of interests

The anti-Trop2 CAR technology described in this report is subject to patent protection (patent no. ZL2022 10431513.2). L.K. and Z.Y. are listed as inventors on the said patent.

## Declaration of generative AI and AI-assisted technologies in the manuscript preparation process

During the preparation of this work, the authors used ChatGPT to assist with language polishing. All content generated using this tool was carefully reviewed and edited by the authors, who take full responsibility for the final version of the manuscript.

## Supplemental information

Document S1. Figures S1 and S2 and Tables S1–S5

Document S2. Article plus supplemental information

## References

[1] Mittal N., Nagar S., Patil A., Rane S.U., Nisarga P., Rabade K., Janu A., Nair D., Thiagarajan S., Laskar S.G. (2025). SMARCB1-deficient Sinonasal Carcinoma: Expanding the Pathologic Spectrum With a Series of 32 Cases. Am. J. Surg. Pathol..

[2] Meerwein C.M., Mauthe T., Soyka M.B., Holzmann D. (2025). Current concepts in sinonasal tumors: from pretherapeutic assessment to patient-tailored treatment. Rhinology.

[3] Chitguppi C., Rabinowitz M.R., Johnson J., Bar-Ad V., Fastenberg J.H., Molligan J., Berman E., Nyquist G.G., Rosen M.R., Evans J.E., Mardekian S.K. (2020). Loss of SMARCB1 Expression Confers Poor Prognosis to Sinonasal Undifferentiated Carcinoma. J. Neurol. Surg. B Skull Base.

[4] Agaimy A. (2022). Proceedings of the North American Society of Head and Neck Pathology, Los Angeles, CA, March 20, 2022: SWI/SNF-deficient Sinonasal Neoplasms: An Overview. Head Neck Pathol..

[5] Zhao S., Du H., Zhang Z., Yang J., Zhou Y., Xiao G., Ma H., Lan C., Liang J., Yang K., Wen L. (2025). SMARCB1/INI-1-Deficient sinonasal carcinoma demonstrates a poor prognosis but favorable clinical outcomes after PD-1/PD-L1 inhibitor therapy: A case series. Sci. Prog..

[6] Li J., Liu C., Zhang P., Shen L., Qi C. (2025). Optimizing CAR T cell therapy for solid tumours: a clinical perspective. Nat. Rev. Clin. Oncol..

[7] Li W., Zhao D., Chen M., Yan X. (2025). Emerging advances in CAR-T therapy for solid tumors: latest clinical trial updates from 2025 ASCO annual meeting. J. Hematol. Oncol..

[8] Zhang X., Xiao H., Na F., Sun J., Guan Q., Liu R., Cai L., Li H., Zhao M. (2025). Evolution of TROP2: Biological insights and clinical applications. Eur. J. Med. Chem..

[9] Liu X., Ma L., Li J., Sun L., Yang Y., Liu T., Xing D., Yan S., Zhang M. (2024). Trop2-targeted therapies in solid tumors: advances and future directions. Theranostics.

[10] Pieniążek M., Kubeczko M., Las-Jankowska M., Polakiewicz-Gilowska A., Łacko A., Jarząb M., Bielčiková Z., Pacholczak-Madej R., Püsküllüoğlu M. (2025). Sacituzumab Govitecan initial dose reduction in polish patients with metastatic triple-negative breast cancer: impact on efficacy and safety. Cancer Chemother. Pharmacol..

[11] Brea E.J., Baldacci S., Savage N., Facchinetti F., Hinchey C., Chakravarti S., Mottram A., Ngo K., Vo H., Leeper B.A. (2025). Systematic Engineering of TROP2-Targeted CAR T-Cell Therapy Overcomes Resistance Pathways in Solid Tumors. Cancer Immunol. Res..

[12] Ansab M., Rath S., Alam U., Burhan M., Siddiqui H.T., Desai A. (2026). TROP-2-directed antibody-drug conjugates in advanced NSCLC: A systematic review and meta-analysis of efficacy, safety, and reconstructed survival outcomes. Crit. Rev. Oncol. Hematol..

[13] Anders P., Sextro M., Lingelbach K., Standvoss K., Pandhe S., Ghosh S., Böhm C., Tietz S., Krupar R., Tharun L. (2026). ADC target profiling in NSCLC: Generalizable AI separates TROP-2 and cMET phenotypes. Clin. Cancer Res..

[14] Luo W., Li C., Zhang Y., Du M., Kou H., Lu C., Mei H., Hu Y. (2022). Adverse effects in hematologic malignancies treated with chimeric antigen receptor (CAR) T cell therapy: a systematic review and Meta-analysis. BMC Cancer.

[15] Li J., Chen H., Xu C., Hu M., Li J., Chang W. (2024). Systemic toxicity of CAR-T therapy and potential monitoring indicators for toxicity prevention. Front. Immunol..

[16] Chen J., Qiu S., Li W., Wang K., Zhang Y., Yang H., Liu B., Li G., Li L., Chen M. (2023). Tuning charge density of chimeric antigen receptor optimizes tonic signaling and CAR-T cell fitness. Cell Res..

[17] Qiu S., Chen J., Wu T., Li L., Wang G., Wu H., Song X., Liu X., Wang H. (2024). CAR-Toner: an AI-driven approach for CAR tonic signaling prediction and optimization. Cell Res..

[18] Landoni E., Fucá G., Wang J., Chirasani V.R., Yao Z., Dukhovlinova E., Ferrone S., Savoldo B., Hong L.K., Shou P. (2021). Modifications to the Framework Regions Eliminate Chimeric Antigen Receptor Tonic Signaling. Cancer Immunol. Res..

[19] Shukla D., Gabunia K., McGettigan S.E., Patel P.R., Christensen S., Fan T.J., Song D., Luo Y., Wang Y., Wang H. (2025). CAR Binders Affect CAR T-cell Tonic Signaling, Durability, and Sensitivity to Target. Cancer Immunol. Res..

[20] Huang Y., Wang H. (2025). Tonic signaling in CAR-T therapy: the lever long enough to move the planet. Front. Med..

[21] Barden M., Elsenbroich P.R., Haas V., Ertelt M., Pervan P., Velas L., Gergely B., Szöőr Á., Harrer D.C., Bezler V. (2024). Integrating binding affinity and tonic signaling enables a rational CAR design for augmented T cell function. J. Immunother. Cancer.

