# Supplementary material for: First-in-human anti-Trop2 CAR-T cell therapy in refractory INI-1/BRM-deficient sinonasal carcinoma: A case report
Source: Mol Ther Oncol. 2026 Oct 14;34(4):201366. doi: 10.1016/j.omton.2026.201366 (PMC13769490; doi:10.1016/j.omton.2026.201366)
Supplement: Supplementary file 1 [file mmc1.pdf]

OMTON, Volume *34*

Supplemental information

First-in-human anti-Trop2 CAR-T cell therapy in refractory INI-1/BRM-deficient sinonasal carcinoma: A case report

Guangfei Li, Yuting Lai, Liang Wang, Hongjia Zhu, Min Chen, Jifang Wang, Taomin Huang, Liqing Kang, Nan Xu, Minghao Li, Xiaoyan Lou, Zhou Yu, Wei Wang, Minghao Fan, Jiali Wu, Haitao Wu, Lei Yu, Peijie He, Hongmeng Yu, and Jian Chen

Materials and Methods

CAR vector design

The CAR construct incorporates a humanized anti-Trop2 single-chain variable fragment (scFv) targeting Trop2, a clinically validated target. The construct contains a 4-1BB co-stimulatory domain and a CD3ζ signaling domain. The CAR vector was co-transfected with three packaging plasmids into HEK 293T cells, and the resulting lentiviruses were harvested, purified by chromatography, and stored immediately at −80°C.

Manufacturing

Peripheral blood mononuclear cells were isolated from patients by density gradient centrifugation, and T cells were enriched by positive selection using magnetic bead separation (Miltenyi Biotec, 200-070-132 and 200-070-115). The enriched T cells were transduced with recombinant lentiviral vectors for 48 h and subsequently cultured for 12–14 days in AIM-V medium (Gibco, A3021002) supplemented with cytokines and autologous human serum. Bacterial, fungal, and mycoplasmal contamination testing was performed throughout CAR-T cell preparation and on the day of final product release.

Transduction efficiency

A total of 1×10^6 cells were incubated with FITC-labeled recombinant protein L (ACRO, RPL-PF141) and PE/Cyanine7 anti-human CD3 Antibody (BioLegend, 300420) at room temperature for 20 min, then washed with PBS, resuspended, and analyzed by flow cytometry.

CAR-T cell Phenotyping

A total of 1×10^6 cells were incubated with T-cell subtype detection reagents at room temperature for 30 min, washed, and analyzed by flow cytometry. T-cell subpopulations were stained with an Alexa Fluor 700-conjugated anti-CD4 monoclonal antibody (eBioscience, 56-0049-42) and an APC/Cyanine7-conjugated anti-human CD8

antibody (BioLegend, 344714). A PE-conjugated anti-human CD197 (CCR7) antibody (BioLegend, 353204), a PerCP/Cyanine5.5-conjugated anti-human CD45RA antibody (BioLegend, 304122), a PE-Cyanine7-conjugated CD127 monoclonal antibody (eBioscience, 25-1278-42), and a FITC-conjugated anti-human CD25 antibody (BD Pharmingen, 555431) were also used. Antibodies used to characterize T lymphocyte exhaustion were as follows: an Alexa Fluor 700-conjugated anti-CD4 monoclonal antibody (eBioscience, 56-0049-42), an APC/Cyanine7-conjugated anti-human CD8 antibody (BioLegend, 344714), a FITC-conjugated anti-CD223 (LAG-3) monoclonal antibody (eBioscience, 11-2239-42), a PE-conjugated mouse anti-human TIM-3 (CD366) antibody (BioLegend, 345006), and a PE-Cyanine7-conjugated anti-CD152 (CTLA-4) monoclonal antibody (eBioscience, 25-1529-42). All flow cytometry antibodies were used according to the manufacturers' instructions.

Release criteria

The final CAR-T cell product was released based on a predefined panel of quality control tests. Appearance was required to be a pale yellow, non-transparent solid that resuspended as a pale yellow suspension upon reconstitution. Osmolality had to be within 1000–1500 mOsmol/kg, and pH within 6.00–7.50. Cell viability was required to be ≥70.00%, with viable cell density reported. The proportion of $CD3^+$ T cells had to be ≥90.00%, and that of $CD3^+CAR^+$ cells ≥5.00%. The proportions of $CD3^+CD4^+$, $CD3^+CD8^+$, CAR^+CD4^+, and CAR^+CD8^+ cells were reported. Cytotoxicity had to be ≥5.00%, and the proportion of $CD19^+$ B cells ≤1.00%. Mycoplasma and sterility testing (rapid method) were required to be negative, and bacterial endotoxin had to be ≤5.000 EU/mL. Only products meeting all criteria were released for clinical use.

Pharmacokinetics

Peripheral blood samples were collected at predefined time points, and CAR-T pharmacokinetic parameters were assessed by qPCR and flow cytometry as described below.

qPCR quantification of CAR transgene copies

Genomic DNA was extracted from 700 μL whole blood using a blood DNA extraction kit (Tiangen, DP348-03) according to the manufacturer's instructions and diluted to 10 ng/μL. Absolute quantification of CAR transgene copies was performed by qPCR using the following primers: forward, 5′-CGCTATGTGGATACGCTGCT-3′; reverse, 5′-GTTGCGTCAGCAAACACAGT-3′. A standard curve was generated by 10-fold serial dilution of a DNA quantitative reference standard. Primers and probe were synthesized by SYNBIO TECHNOLOGIES. Each 25 μL reaction contained 20 μL qPCR master mix (12.5 μL 2× AceQ Probe Master Mix, 0.5 μL forward primer, 0.5 μL reverse primer, 1.0 μL FAM-labeled probe, and 5.5 μL DNase/RNase-free water) and 5 μL of standard, no-template control, or sample. The amplification program consisted of 95 °C for 5 min, followed by 40 cycles of 95 °C for 15 s and 60 °C for 50 s, with fluorescence acquisition at the extension step. CAR copies were calculated from the standard curve and normalized to input DNA. All samples were run in duplicate.

Flow cytometric detection of CAR-positive cells

Peripheral blood samples were collected in EDTA tubes, and white blood cells were counted. Approximately 1 × 10^6 white blood cells per tube were used. Red blood cells were lysed with 1× RBC Lysis Buffer (Thermo, 2410277) at a ratio of 8:1, incubated at room temperature in the dark for 10 min, and washed twice with PBS (Corning, 21-040-CVC). Cells were resuspended in 150 μL PBS and divided into a sample tube and a control tube. Sample tube cells were incubated with His-tagged human Trop2/TACSTD2 protein (ACRO, TR2-H5223) at 4 °C for 45 min, washed, and then stained with Brilliant Violet 785™ anti-human CD45 antibody (BioLegend, 304048), APC/Cyanine7 anti-human CD3 antibody (BioLegend, 300318), and HIS-PE secondary antibody (BioLegend, 362603) at room temperature for 30 min in the dark. After incubation, cells were washed with PBS, resuspended, and analyzed by flow cytometry.

Multiplexed bead-based immunoassay

The patient’s blood samples were subjected to cytokine profiling using a 12-plex human

cytokine detection kit (Catalog No. 191217; Hangzhou R&D Biological Technology Co., Ltd.). The inflammatory mediators quantified were IL-1β, IL-2, IL-4, IL-5, IL-6, IL-8, IL-10, IL-12, IL-17, TNF-α and IFN-γ. The assay was based on a bead-based multiplex flow cytometry immunoassay, utilizing microsphere sandwich immunofluorescence in conjunction with ELISA principles.
