# Supplementary material for: First-in-human anti-Trop2 CAR-T cell therapy in refractory INI-1/BRM-deficient sinonasal carcinoma: A case report
Source: Mol Ther Oncol. 2026 Oct 14;34(4):201366. doi: 10.1016/j.omton.2026.201366 (PMC13769490; doi:10.1016/j.omton.2026.201366)
Supplement: Supplementary file 2 [file mmc2.pdf]

Molecular Therapy
Oncology
Case report

First-in-human anti-Trop2 CAR-T cell therapy in refractory INI-1/BRM-deficient sinonasal carcinoma: A case report

Guangfei Li,[1,7] Yuting Lai,[1,7] Liang Wang,[1,7] Hongjia Zhu,[2,3,7] Min Chen,[1,7] Jifang Wang,[4] Taomin Huang,[5] Liqing Kang,[3] Nan Xu,[3] Minghao Li,[3] Xiaoyan Lou,[3] Zhou Yu,[3] Wei Wang,[3] Minghao Fan,[3] Jiali Wu,[3] Haitao Wu,[1,6] Lei Yu,[3] Peijie He,[1] Hongmeng Yu,[1] and Jian Chen[1,6]

[1]ENT Institute and Department of Otorhinolaryngology, Eye & ENT Hospital, Fudan University, Shanghai 200031, China; [2]School of Pharmacy, East China Normal University, Shanghai 200062, China; [3]Shanghai Unicar-Therapy Bio-medicine Technology Co., Ltd, Shanghai 200000, China; [4]Department of Nursing, Eye & ENT Hospital, Fudan University, Shanghai 200031, China; [5]Department of Pharmacy, Eye & ENT Hospital, Fudan University, Shanghai 200031, China; [6]Shanghai Key Laboratory of Gene Editing and Cell Therapy for Rare Diseases, Fudan University, Shanghai 200031, China

INI-1-deficient sinonasal carcinoma is an aggressive malignancy with a dismal prognosis and no established salvage therapies. While trophoblast cell-surface antigen 2 (Trop2) is a promising immunotherapeutic target, its clinical utility in sinonasal cancer remains unexplored. We report the first-in-human application of anti-Trop2 CAR-T cell therapy in a 61-year-old male with refractory INI-1/BRM-deficient sinonasal carcinoma. Following lymphodepletion, the CAR-T cells demonstrated robust *in vivo* expansion, peaking within 14 days and coinciding with transient cytokine elevation. Toxicities were manageable but included grade 2 cytokine release syndrome and grade 3 hepatotoxicity that required multiple sessions of plasma exchange. Notably, MRI at day 78 post-infusion revealed dramatic tumor regression with extensive necrosis and liquefaction, which correlated with the complete resolution of intractable orbital pain. This single-case observation provides preliminary proof-of-concept for the safety and anti-tumor activity of Trop2-targeted CAR-T cells in refractory sinonasal malignancies, though further studies are needed to confirm durability and generalizability.

INTRODUCTION

Sinonasal malignancies, though accounting for only 3%–5% of all head and neck tumors, represent a formidable clinical challenge characterized by aggressive local invasion and profound resistance to conventional multimodal therapies.[1,2] Within this heterogeneous spectrum, carcinomas driven by the deficiency of the switch/sucrose non-fermentable (SWI/SNF) chromatin, most frequently through the biallelic loss of INI-1 (SMARCB1) and/or BRM (SMARCA2), exhibit an undifferentiated histology and an exceptional lydismal-prognosis.[3,4] For patients with recurrent or metastatic disease, no standardized targeted or immunotherapeutic regimens have been established, and the 3-year overall survival rate remains a modest 51.8%.[5]

Chimeric antigen receptor (CAR)-T cell therapy offers a potential paradigm shift for such intractable solid tumors.[6] A pivotal requirement for this cellular approach is the identification of tumor-associated antigens that are stably and abundantly expressed on malignant cells while maintaining an acceptable safety profile.[7] Trophoblast cell surface antigen 2 (Trop2), a transmembrane glycoprotein overexpressed across a wide range of epithelial cancers, has emerged as a clinically validated target that drives tumor proliferation and therapeutic resistance.[8,9] While Trop2-directed antibody-drug conjugates have entered clinical practice, anti-Trop2 CAR-T cells are only recently being explored, with early-phase trials suggesting promising activity in head and neck squamous cell carcinoma.[10,11] However, the clinical utility of Trop2-targeted cellular therapy in sinonasal malignancies remains unexplored.

Here, we report the first clinical application of autologous anti-Trop2 CAR-T cell therapy in a patient with treatment-refractory sinonasal carcinoma harboring concomitant INI-1/BRM deficiency and high

Received 27 August 2026; accepted 13 October 2026;
https://doi.org/10.1016/j.omton.2026.201366.

[7]These authors contributed equally

Correspondence: Lei Yu, Shanghai Unicar-Therapy Bio-medicine Technology Co., Ltd, Shanghai 200000, China.
E-mail: ylyh188@163.com
Correspondence: Peijie He, ENT Institute and Department of Otorhinolaryngology, Eye & ENT Hospital, Fudan University, Shanghai 200031, China.
E-mail: hepj2002@sina.com
Correspondence: Hongmeng Yu, ENT Institute and Department of Otorhinolaryngology, Eye & ENT Hospital, Fudan University, Shanghai 200031, China.
E-mail: hongmengyush@163.com
Correspondence: Jian Chen, ENT Institute and Department of Otorhinolaryngology, Eye & ENT Hospital, Fudan University, Shanghai 200031, China.
E-mail: chenj15@fudan.edu.cn

© 2026 The Authors.
Published by Elsevier Inc. on behalf of The American Society of Gene and Cell Therapy.
This is an open access article under the CC BY license (http://creativecommons.org/licenses/by/4.0/).

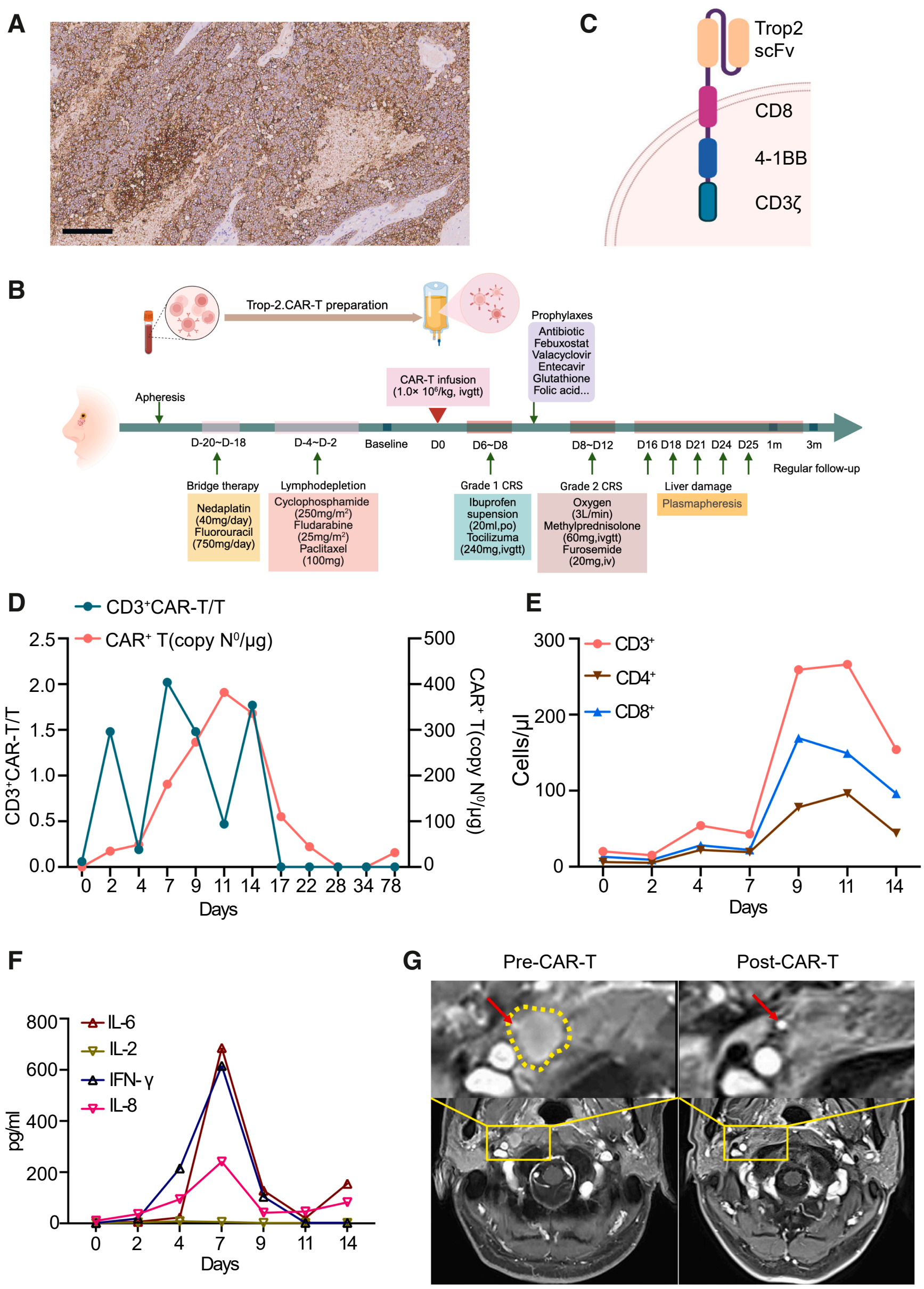

Figure 1. Treatment schema, CAR-T expansion, and anti-tumor response following Trop-2 CAR-T therapy

(A) Tumor immunohistochemistry confirming high Trop-2 expression. (B) Schematic overview of the treatment procedures. (C) CAR construct comprising Trop-2 scFv, CD8 hinge/transmembrane domain, 4-1BB costimulatory domain, and CD3ζ signaling domain. (D) Peripheral expansion of CAR-T cells, shown as $CD3^{+}$CAR-T frequency and transgene copy number, peaking within the first 2 weeks post-infusion. (E) Dynamics of circulating T cell subsets ($CD3^{+}$, $CD4^{+}$, and $CD8^{+}$), demonstrating transient expansion consistent with CAR-T proliferation. (F) Serum cytokine kinetics (IL-6, IL-2, IFN-γ, and IL-8), with a sharp peak around day 7 corresponding to CRS onset. (G) MRI evaluation showing marked tumor regression at day 78 compared with baseline. (Scale bars, 50 μm).

Trop2 expression. We detail the therapeutic feasibility, robust *in vivo* expansion, and remarkable tumor regression achieved in this ultra-rare and aggressive disease entity, providing preliminary proof of concept for Trop2 as a viable therapeutic vulnerability in sinonasal malignancies.

Patient details and clinical findings

In March 2024, a 61-year-old male presented with right nasal obstruction and blood-tinged discharge and underwent endoscopic sinonasal tumor resection. Postoperative pathology confirmed "INI-1-deficient carcinoma of the right nasal cavity." He subsequently received two cycles of chemotherapy in April 2024, followed by a second radical resection of the right sinonasal tumor in June 2024. In July 2024, he underwent radiotherapy (50 fractions) with concurrent chemotherapy. Despite this aggressive multimodal approach, MRI on March 21, 2025, revealed a new soft tissue mass in the right surgical cavity and ethmoid sinus region, indicating local recurrence. On March 31, 2025, the patient underwent a comprehensive salvage surgery at our center, including endoscopic sinonasal tumor resection, endoscopic skull base lesion resection, complete sinusotomy, endoscopic dacryocystorhinostomy, and right orbital decompression. Postoperatively, he received two additional cycles of chemotherapy from April to June 2025. However, the disease continued to progress, and the patient was clinically diagnosed with advanced refractory relapsed INI-1-deficient sinonasal carcinoma. A repeat biopsy during this period confirmed SWI/SNF complex deficiency with concomitant loss of INI-1 and BRM, along with extensive bone invasion. Immunohistochemistry showed strong and diffuse Trop2 expression (Figure 1A), providing the rationale for Trop2-targeted therapy. Additional methodological details are provided in the supplemental information.

The patient was enrolled in the first cohort of an investigator-initiated phase 1 clinical trial (NCT06614686) employing a standard 3 + 3 dose-escalation design. As the first-treated patient, he received the lowest planned dose level (1.0×10^{6} cells/kg), and thus this report reflects the initial clinical experience with this anti-Trop2 CAR-T product. The trial was approved by the institutional ethics committee.

After obtaining informed consent, the patient underwent leukapheresis on July 10, 2025 (Figure 1B). During CAR-T manufacturing

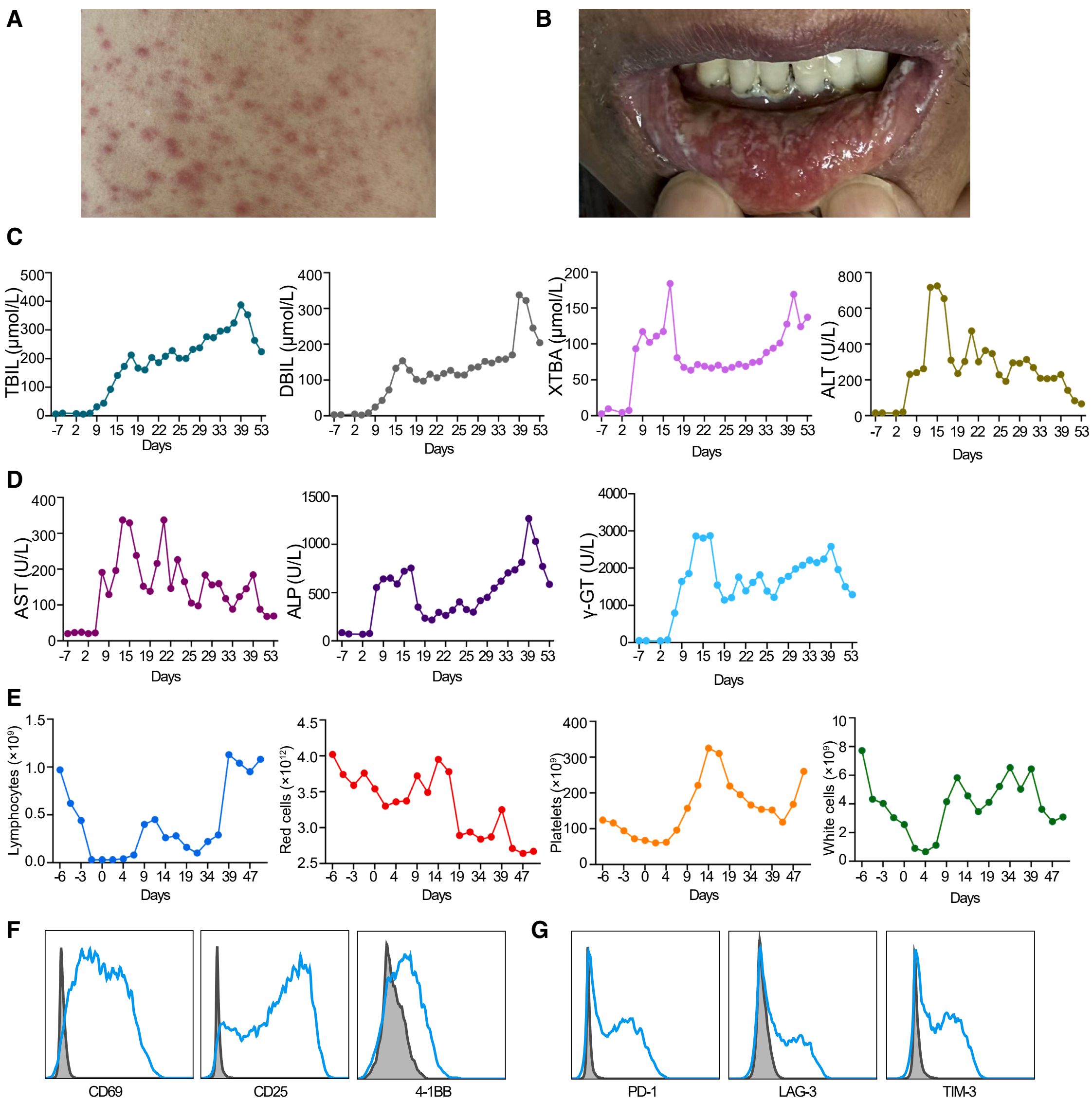

Figure 2. Manageable toxicities and dynamic immune-related adverse events following CAR-T therapy

(A) Diffuse cutaneous rash observed post-infusion. (B) Oral mucosal ulceration as a manifestation of treatment-related toxicity. (C) Liver function parameters (TBIL, DBIL, TBA, and ALT) demonstrating progressive hepatotoxicity after CAR-T infusion. (D) Additional hepatic markers (AST, ALP, and γ-GT) confirming mixed hepatocellular-cholestatic injury. (E) Longitudinal blood counts showing post-lymphodepletion cytopenias followed by gradual hematologic recovery. (F) Flow cytometric analysis showing the tonic signaling of anti-Trop2 CAR-T cells during *ex vivo* expansion in the absence of antigen stimulation on day 14. (G) Increased expression of exhaustion markers (PD-1, TIM-3, and LAG-3) in anti-Trop2 CAR-T cells during *ex vivo* expansion on day 14.

(July 10–August 4), the patient received bridging chemotherapy with nedaplatin plus fluorouracil from July 15 to July 17, 2025, to stabilize tumor burden (nedaplatin 40 mg/day and fluorouracil 0.75 g/day, both administered intravenously once daily for 3 consecutive days). A single intravenous dose of 1.0×10^6 anti-Trop2 CAR-T cells/kg was infused on August 4, 2025 (Figure 1C). The infusion was followed by a transient *in vivo* expansion of the CAR-T cells (Figure 1D). Notably, both $CD4^+$ and $CD8^+$ T cell subsets expanded, with a predominant rise in the $CD8^+$ cytotoxic compartment (Figure 1E). The cellular expansion coincided with a transient elevation of pro-inflammatory cytokines, including IL-6, IFN-γ, and IL-8, which peaked around day 7 (Figure 1F).

Clinically, notwithstanding the limited longitudinal persistence of CAR-T cells in the peripheral circulation, the patient achieved a rapid and profound therapeutic response. Within only one week of infusion, there was a significant and rapid relief of the previously intractable orbital pain, which completely resolved by the second

week. This early symptomatic improvement was further substantiated by definitive radiological evidence at day 78 post-treatment. MRI evaluation revealed a remarkable response characterized by substantial tumor regression, extensive central necrosis, and liquefaction. Notably, the relief of the tumor's mass effect led to the clear demarcation of previously obscured vascular structures, providing robust evidence of a potent anti-tumor effect that persisted well beyond the peak of systemic CAR-T expansion (Figure 1G). No additional anti-cancer therapies (chemotherapy, radiotherapy, or other immunotherapies) were administered after CAR-T cell infusion. The patient received only supportive care and management of adverse events as described in the following text. Therefore, the observed tumor response is primarily attributed to CAR-T cell therapy.

The treatment was associated with manageable toxicities. On day 7, coinciding with peak cytokine levels and CAR-T expansion, the patient developed a high fever and subsequent hypoxia with pulmonary edema. These symptoms, consistent with grade 2 cytokine release syndrome (CRS), were promptly reversed following treatment with tocilizumab, corticosteroids, and supportive diuretics. A self-limiting diffuse erythematous rash appeared on day 2 and resolved by day 14 (Figure 2A). An oral fungal infection observed on day 12 was successfully managed with antifungal therapy (Figure 2B). Hepatotoxicity was the most significant adverse event. Liver enzymes and bilirubin rose from day 8, reaching grade 3 by day 13 (Figures 2C and 2D). Despite hepatoprotective agents, the patient required multiple sessions of plasma exchange (five sessions over two weeks) to manage progressive hyperbilirubinemia and elevated transaminases. Liver function eventually stabilized without progressing to liver failure, but recovery was gradual. Hematologic recovery followed the expected nadir after lymphodepletion (Figure 2E). Phenotypic analyses during *ex vivo* expansion showed tonic signaling (Figure 2F) and increased exhaustion markers (PD-1, TIM-3, and LAG-3) by day 14 (Figure 2G), suggesting antigen-independent tonic signaling that may have limited *in vivo* persistence.

DISCUSSION

This single-case report provides initial clinical evidence that anti-Trop2 CAR-T therapy can induce rapid tumor regression in refractory INI-1/BRM-deficient sinonasal carcinoma. The response is notable given the aggressive nature of this malignancy and the failure of multiple prior modalities. Trop2 is validated as a target by antibody-drug conjugates,[12,13] and our observation extends this to cellular immunotherapy. However, as a single case with short follow-up, no definitive conclusions on efficacy can be drawn.

Safety remains a key concern. Grade 2 CRS and grade 3 hepatotoxicity occurred, the latter required multiple plasma exchange sessions, highlighting the need for close monitoring. Liver injury may be multifactorial, including cytokine-mediated inflammation or on-target/off-tumor effects from low-level Trop2 expression in biliary or hepatic tissues.[14,15] These events were reversible with standard management, suggesting acceptable tolerability at this dose.

A notable finding is the dissociation between robust early tumor necrosis and limited CAR-T persistence. The patient achieved significant tumor reduction by day 78, yet CAR-T cells were barely detectable after day 17. This limited persistence may be due to tonic signaling-induced exhaustion, as our *ex vivo* data show spontaneous upregulation of activation and exhaustion markers without antigen stimulation (Figures 2F and 2G). Previous work has linked CAR tonic signaling to electrostatic interactions of the single-chain variable fragment (scFv), quantified by the positively charged patches (PCP) score.[16,17] Although this CAR has a PCP score of 33 (below the optimal ∼40), it exhibited high tonic signaling, suggesting non-electrostatic mechanisms such as scFv stability or framework integrity.[18–21] Engineering improvements are needed to reduce tonic signaling and enhance persistence.

In conclusion, this first report of anti-Trop2 CAR-T therapy in INI-1/BRM-deficient sinonasal carcinoma demonstrates feasibility and a promising initial response but also highlights the challenge of limited CAR-T persistence. Prospective trials with larger cohorts and modified CAR designs are required to assess long-term efficacy and safety.

DATA AND CODE AVAILABILITY

Detailed preclinical validation of this CAR construct is available in a companion manuscript currently under review. These data can be made available to the editor and reviewers confidentially if required.

ACKNOWLEDGMENTS

We thank all the participants for their generous support for the current study. This study was supported by the Shanghai Hospital Development Center Foundation (SHDC12025122), the National Natural Science Foundation of China (82471150 to J.C.), the Shanghai Rising-Star Program (23QA1400900 to J.C.), China University Industry-Academia-Research Innovation Fund (2024DR033 to P.H.), as well as the Shanghai Clinical Research Center for Cell Therapy (23J41900100). This study was approved by the Institutional Review Board/Ethics Committee of Eye & ENT Hospital, Fudan University (approval no. 2024145). The trial is registered at ClinicalTrials.gov (NCT06614686). Written informed consent was obtained from the patient for participation in the study. A copy of the written informed consent is available for review by the editorial office upon request.

AUTHOR CONTRIBUTIONS

The study was designed by P.H., H.Y., and J.C.; clinical data and patient care were provided by J. Wang., T.H., P.H., H.Y., J.C., and L.Y.; formal analyses were performed by G.L., Y.L., L.W., H.Z., and M.C.; figures were created by G.L., Y.L., and H.Z.; data were interpreted by G.L., Y.L., L.W., H.Z., M.C., J. Wang, T.H., L.K., N.X., M.L., X.L., Z.Y., W.W., M.F., J. Wu, L.Y., P.H., H.Y., and J.C.; resources were provided by J. Wang, T.H., L.K., N.X., M.L., X.L., Z.Y., W.W., M.F., J. Wu, L.Y., P.H., H.Y., and J.C.; original draft was written by G.L., Y.L., L.W., H.Z., M.C., P.H., H.Y., and J.C.; all authors critically reviewed and edited the manuscript; the study was supervised by P.H., H.Y., L.Y., and J.C.; the guarantors of the study are P.H., H.Y., and J.C.

DECLARATION OF INTERESTS

The anti-Trop2 CAR technology described in this report is subject to patent protection (patent no. ZL2022 10431513.2). L.K. and Z.Y. are listed as inventors on the said patent.

DECLARATION OF GENERATIVE AI AND AI-ASSISTED TECHNOLOGIES IN THE MANUSCRIPT PREPARATION PROCESS

During the preparation of this work, the authors used ChatGPT to assist with language polishing. All content generated using this tool was carefully reviewed and edited by the authors, who take full responsibility for the final version of the manuscript.

SUPPLEMENTAL INFORMATION

Supplemental information can be found online at https://doi.org/10.1016/j.omton.2026.201366.

REFERENCES

1. Mittal, N., Nagar, S., Patil, A., Rane, S.U., Nisarga, P., Rabade, K., Janu, A., Nair, D., Thiagarajan, S., Laskar, S.G., et al. (2025). SMARCB1-deficient Sinonasal Carcinoma: Expanding the Pathologic Spectrum With a Series of 32 Cases. Am. J. Surg. Pathol. *49*, 381–393. https://doi.org/10.1097/PAS.0000000000002364.
2. Meerwein, C.M., Mauthe, T., Soyka, M.B., and Holzmann, D. (2025). Current concepts in sinonasal tumors: from pretherapeutic assessment to patient-tailored treatment. Rhinology *63*, 534–542. https://doi.org/10.4193/Rhin25.073.
3. Chitguppi, C., Rabinowitz, M.R., Johnson, J., Bar-Ad, V., Fastenberg, J.H., Molligan, J., Berman, E., Nyquist, G.G., Rosen, M.R., Evans, J.E., and Mardekian, S.K. (2020). Loss of SMARCB1 Expression Confers Poor Prognosis to Sinonasal Undifferentiated Carcinoma. J. Neurol. Surg. B Skull Base *81*, 610–619. https://doi.org/10.1055/s-0039-1693659.
4. Agaimy, A. (2022). Proceedings of the North American Society of Head and Neck Pathology, Los Angeles, CA, March 20, 2022: SWI/SNF-deficient Sinonasal Neoplasms: An Overview. Head Neck Pathol. *16*, 168–178. https://doi.org/10.1007/s12105-022-01416-x.
5. Zhao, S., Du, H., Zhang, Z., Yang, J., Zhou, Y., Xiao, G., Ma, H., Lan, C., Liang, J., Yang, K., and Wen, L. (2025). SMARCB1/INI-1-Deficient sinonasal carcinoma demonstrates a poor prognosis but favorable clinical outcomes after PD-1/PD-L1 inhibitor therapy: A case series. Sci. Prog. *108*, 368504251315075. https://doi.org/10.1177/00368504251315075.
6. Li, J., Liu, C., Zhang, P., Shen, L., and Qi, C. (2025). Optimizing CAR T cell therapy for solid tumours: a clinical perspective. Nat. Rev. Clin. Oncol. *22*, 953–968. https://doi.org/10.1038/s41571-025-01075-1.
7. Li, W., Zhao, D., Chen, M., and Yan, X. (2025). Emerging advances in CAR-T therapy for solid tumors: latest clinical trial updates from 2025 ASCO annual meeting. J. Hematol. Oncol. *18*, 104. https://doi.org/10.1186/s13045-025-01760-9.
8. Zhang, X., Xiao, H., Na, F., Sun, J., Guan, Q., Liu, R., Cai, L., Li, H., and Zhao, M. (2025). Evolution of TROP2: Biological insights and clinical applications. Eur. J. Med. Chem. *296*, 117863. https://doi.org/10.1016/j.ejmech.2025.117863.
9. Liu, X., Ma, L., Li, J., Sun, L., Yang, Y., Liu, T., Xing, D., Yan, S., and Zhang, M. (2024). Trop2-targeted therapies in solid tumors: advances and future directions. Theranostics *14*, 3674–3692. https://doi.org/10.7150/thno.98178.
10. Pieniążek, M., Kubeczko, M., Las-Jankowska, M., Polakiewicz-Gilowska, A., Łacko, A., Jarząb, M., Bielčiková, Z., Pacholczak-Madej, R., and Püsküllüoğlu, M. (2025). Sacituzumab Govitecan initial dose reduction in polish patients with metastatic triple-negative breast cancer: impact on efficacy and safety. Cancer Chemother. Pharmacol. *95*, 97. https://doi.org/10.1007/s00280-025-04823-3.
11. Brea, E.J., Baldacci, S., Savage, N., Facchinetti, F., Hinchey, C., Chakravarti, S., Mottram, A., Ngo, K., Vo, H., Leeper, B.A., et al. (2025). Systematic Engineering of TROP2-Targeted CAR T-Cell Therapy Overcomes Resistance Pathways in Solid Tumors. Cancer Immunol. Res. *13*, 1749–1763. https://doi.org/10.1158/2326-6066.CIR-25-0527.
12. Ansab, M., Rath, S., Alam, U., Burhan, M., Siddiqui, H.T., and Desai, A. (2026). TROP-2-directed antibody-drug conjugates in advanced NSCLC: A systematic review and meta-analysis of efficacy, safety, and reconstructed survival outcomes. Crit. Rev. Oncol. Hematol. *218*, 105093. https://doi.org/10.1016/j.critrevonc.2025.105093.
13. Anders, P., Sextro, M., Lingelbach, K., Standvoss, K., Pandhe, S., Ghosh, S., Böhm, C., Tietz, S., Krupar, R., Tharun, L., et al. (2026). ADC target profiling in NSCLC: Generalizable AI separates TROP-2 and cMET phenotypes. Clin. Cancer Res. *32*, 2765–2786. https://doi.org/10.1158/1078-0432.Ccr-25-4513.
14. Luo, W., Li, C., Zhang, Y., Du, M., Kou, H., Lu, C., Mei, H., and Hu, Y. (2022). Adverse effects in hematologic malignancies treated with chimeric antigen receptor (CAR) T cell therapy: a systematic review and Meta-analysis. BMC Cancer *22*, 98. https://doi.org/10.1186/s12885-021-09102-x.
15. Li, J., Chen, H., Xu, C., Hu, M., Li, J., and Chang, W. (2024). Systemic toxicity of CAR-T therapy and potential monitoring indicators for toxicity prevention. Front. Immunol. *15*, 1422591. https://doi.org/10.3389/fimmu.2024.1422591.
16. Chen, J., Qiu, S., Li, W., Wang, K., Zhang, Y., Yang, H., Liu, B., Li, G., Li, L., Chen, M., et al. (2023). Tuning charge density of chimeric antigen receptor optimizes tonic signaling and CAR-T cell fitness. Cell Res. *33*, 341–354. https://doi.org/10.1038/s41422-023-00789-0.
17. Qiu, S., Chen, J., Wu, T., Li, L., Wang, G., Wu, H., Song, X., Liu, X., and Wang, H. (2024). CAR-Toner: an AI-driven approach for CAR tonic signaling prediction and optimization. Cell Res. *34*, 386–388. https://doi.org/10.1038/s41422-024-00936-1.
18. Landoni, E., Fucá, G., Wang, J., Chirasani, V.R., Yao, Z., Dukhovlinova, E., Ferrone, S., Savoldo, B., Hong, L.K., Shou, P., et al. (2021). Modifications to the Framework Regions Eliminate Chimeric Antigen Receptor Tonic Signaling. Cancer Immunol. Res. *9*, 441–453. https://doi.org/10.1158/2326-6066.CIR-20-0451.
19. Shukla, D., Gabunia, K., McGettigan, S.E., Patel, P.R., Christensen, S., Fan, T.J., Song, D., Luo, Y., Wang, Y., Wang, H., et al. (2025). CAR Binders Affect CAR T-cell Tonic Signaling, Durability, and Sensitivity to Target. Cancer Immunol. Res. *13*, 867–880. https://doi.org/10.1158/2326-6066.CIR-24-1347.
20. Huang, Y., and Wang, H. (2025). Tonic signaling in CAR-T therapy: the lever long enough to move the planet. Front. Med. *19*, 391–408. https://doi.org/10.1007/s11684-025-1130-x.
21. Barden, M., Elsenbroich, P.R., Haas, V., Ertelt, M., Pervan, P., Velas, L., Gergely, B., Szöőr, Á., Harrer, D.C., Bezler, V., et al. (2024). Integrating binding affinity and tonic signaling enables a rational CAR design for augmented T cell function. J. Immunother. Cancer *12*, e010208. https://doi.org/10.1136/jitc-2024-010208.

OMTON, Volume *34*

Supplemental information

First-in-human anti-Trop2 CAR-T cell therapy in refractory INI-1/BRM-deficient sinonasal carcinoma: A case report

Guangfei Li, Yuting Lai, Liang Wang, Hongjia Zhu, Min Chen, Jifang Wang, Taomin Huang, Liqing Kang, Nan Xu, Minghao Li, Xiaoyan Lou, Zhou Yu, Wei Wang, Minghao Fan, Jiali Wu, Haitao Wu, Lei Yu, Peijie He, Hongmeng Yu, and Jian Chen

Materials and Methods

CAR vector design

The CAR construct incorporates a humanized anti-Trop2 single-chain variable fragment (scFv) targeting Trop2, a clinically validated target. The construct contains a 4-1BB co-stimulatory domain and a CD3ζ signaling domain. The CAR vector was co-transfected with three packaging plasmids into HEK 293T cells, and the resulting lentiviruses were harvested, purified by chromatography, and stored immediately at −80°C.

Manufacturing

Peripheral blood mononuclear cells were isolated from patients by density gradient centrifugation, and T cells were enriched by positive selection using magnetic bead separation (Miltenyi Biotec, 200-070-132 and 200-070-115). The enriched T cells were transduced with recombinant lentiviral vectors for 48 h and subsequently cultured for 12–14 days in AIM-V medium (Gibco, A3021002) supplemented with cytokines and autologous human serum. Bacterial, fungal, and mycoplasmal contamination testing was performed throughout CAR-T cell preparation and on the day of final product release.

Transduction efficiency

A total of 1×10^6 cells were incubated with FITC-labeled recombinant protein L (ACRO, RPL-PF141) and PE/Cyanine7 anti-human CD3 Antibody (BioLegend, 300420) at room temperature for 20 min, then washed with PBS, resuspended, and analyzed by flow cytometry.

CAR-T cell Phenotyping

A total of 1×10^6 cells were incubated with T-cell subtype detection reagents at room temperature for 30 min, washed, and analyzed by flow cytometry. T-cell subpopulations were stained with an Alexa Fluor 700-conjugated anti-CD4 monoclonal antibody (eBioscience, 56-0049-42) and an APC/Cyanine7-conjugated anti-human CD8

antibody (BioLegend, 344714). A PE-conjugated anti-human CD197 (CCR7) antibody (BioLegend, 353204), a PerCP/Cyanine5.5-conjugated anti-human CD45RA antibody (BioLegend, 304122), a PE-Cyanine7-conjugated CD127 monoclonal antibody (eBioscience, 25-1278-42), and a FITC-conjugated anti-human CD25 antibody (BD Pharmingen, 555431) were also used. Antibodies used to characterize T lymphocyte exhaustion were as follows: an Alexa Fluor 700-conjugated anti-CD4 monoclonal antibody (eBioscience, 56-0049-42), an APC/Cyanine7-conjugated anti-human CD8 antibody (BioLegend, 344714), a FITC-conjugated anti-CD223 (LAG-3) monoclonal antibody (eBioscience, 11-2239-42), a PE-conjugated mouse anti-human TIM-3 (CD366) antibody (BioLegend, 345006), and a PE-Cyanine7-conjugated anti-CD152 (CTLA-4) monoclonal antibody (eBioscience, 25-1529-42). All flow cytometry antibodies were used according to the manufacturers' instructions.

Release criteria

The final CAR-T cell product was released based on a predefined panel of quality control tests. Appearance was required to be a pale yellow, non-transparent solid that resuspended as a pale yellow suspension upon reconstitution. Osmolality had to be within 1000–1500 mOsmol/kg, and pH within 6.00–7.50. Cell viability was required to be ≥70.00%, with viable cell density reported. The proportion of $CD3^+$ T cells had to be ≥90.00%, and that of $CD3^+CAR^+$ cells ≥5.00%. The proportions of $CD3^+CD4^+$, $CD3^+CD8^+$, CAR^+CD4^+, and CAR^+CD8^+ cells were reported. Cytotoxicity had to be ≥5.00%, and the proportion of $CD19^+$ B cells ≤1.00%. Mycoplasma and sterility testing (rapid method) were required to be negative, and bacterial endotoxin had to be ≤5.000 EU/mL. Only products meeting all criteria were released for clinical use.

Pharmacokinetics

Peripheral blood samples were collected at predefined time points, and CAR-T pharmacokinetic parameters were assessed by qPCR and flow cytometry as described below.

qPCR quantification of CAR transgene copies

Genomic DNA was extracted from 700 μL whole blood using a blood DNA extraction kit (Tiangen, DP348-03) according to the manufacturer's instructions and diluted to 10 ng/μL. Absolute quantification of CAR transgene copies was performed by qPCR using the following primers: forward, 5′-CGCTATGTGGATACGCTGCT-3′; reverse, 5′-GTTGCGTCAGCAAACACAGT-3′. A standard curve was generated by 10-fold serial dilution of a DNA quantitative reference standard. Primers and probe were synthesized by SYNBIO TECHNOLOGIES. Each 25 μL reaction contained 20 μL qPCR master mix (12.5 μL 2× AceQ Probe Master Mix, 0.5 μL forward primer, 0.5 μL reverse primer, 1.0 μL FAM-labeled probe, and 5.5 μL DNase/RNase-free water) and 5 μL of standard, no-template control, or sample. The amplification program consisted of 95 °C for 5 min, followed by 40 cycles of 95 °C for 15 s and 60 °C for 50 s, with fluorescence acquisition at the extension step. CAR copies were calculated from the standard curve and normalized to input DNA. All samples were run in duplicate.

Flow cytometric detection of CAR-positive cells

Peripheral blood samples were collected in EDTA tubes, and white blood cells were counted. Approximately 1×10^6 white blood cells per tube were used. Red blood cells were lysed with 1× RBC Lysis Buffer (Thermo, 2410277) at a ratio of 8:1, incubated at room temperature in the dark for 10 min, and washed twice with PBS (Corning, 21-040-CVC). Cells were resuspended in 150 μL PBS and divided into a sample tube and a control tube. Sample tube cells were incubated with His-tagged human Trop2/TACSTD2 protein (ACRO, TR2-H5223) at 4 °C for 45 min, washed, and then stained with Brilliant Violet 785™ anti-human CD45 antibody (BioLegend, 304048), APC/Cyanine7 anti-human CD3 antibody (BioLegend, 300318), and HIS-PE secondary antibody (BioLegend, 362603) at room temperature for 30 min in the dark. After incubation, cells were washed with PBS, resuspended, and analyzed by flow cytometry.

Multiplexed bead-based immunoassay

The patient’s blood samples were subjected to cytokine profiling using a 12-plex human

cytokine detection kit (Catalog No. 191217; Hangzhou R&D Biological Technology Co., Ltd.). The inflammatory mediators quantified were IL-1β, IL-2, IL-4, IL-5, IL-6, IL-8, IL-10, IL-12, IL-17, TNF-α and IFN-γ. The assay was based on a bead-based multiplex flow cytometry immunoassay, utilizing microsphere sandwich immunofluorescence in conjunction with ELISA principles.
